# Revolutions in Neuroscience: Tool Development

**DOI:** 10.3389/fnsys.2016.00024

**Published:** 2016-03-08

**Authors:** John Bickle

**Affiliations:** ^1^Department of Philosophy and Religion, Mississippi State UniversityMississippi, MS, USA; ^2^Department of Neurobiology and Anatomical Sciences, University of Mississippi Medical CenterJackson, MS, USA

**Keywords:** Thomas Kuhn’s model of scientific revolution, tool development experiments, metascience, gene targeting techniques, optogenetics and DREADDs, motivating problem, hook experiments

## Abstract

Thomas Kuhn’s famous model of the components and dynamics of scientific revolutions is still dominant to this day across science, philosophy, and history. The guiding philosophical theme of this article is that, concerning actual revolutions in neuroscience over the past 60 years, Kuhn’s account is wrong. There have been revolutions, and new ones are brewing, but they do not turn on competing paradigms, anomalies, or the like. Instead, they turn exclusively on the development of new experimental tools. I adopt a metascientific approach and examine in detail the development of two recent neuroscience revolutions: the impact of engineered genetically mutated mammals in the search for causal mechanisms of “higher” cognitive functions; and the more recent impact of optogenetics and designer receptors exclusively activated by designer drugs (DREADDs). The two key metascientific concepts, I derive from these case studies are a revolutionary new tool’s *motivating problem*, and its *initial* and *second-phase* *hook experiments*. These concepts hardly exhaust a detailed metascience of tool development experiments in neuroscience, but they get that project off to a useful start and distinguish the subsequent account of neuroscience revolutions clearly from Kuhn’s famous model. I close with a brief remark about the general importance of molecular biology for a current philosophical understanding of science, as comparable to the place physics occupied when Kuhn formulated his famous theory of scientific revolutions.

## Kuhn’S Model and Neuroscience

Kuhn’s (1962) model of scientific revolution still pervades much discussion in science, philosophy, and history. A dominant paradigm eventually emerges within a scientific field. All practitioners learn it—its practices, models, exemplars, and most prominent applications. Normal science ensues: these resources get developed further and the paradigm gets extended beyond its initial applications. Yet anomalies start to arise—applications which seem intuitively promising for the paradigm, but which prove recalcitrant to absorption. Anomalies pile up, and some practitioners begin to notice patterns among them, which suggest the outlines of a new paradigm. Conservative practitioners hold to the still-dominant paradigm, and continue pursuing normal science; radicals, often younger scientists, flock to the emerging new paradigm, to develop its components and extend its applications. At this stage revolutionary science ensues, and evidence and rational argument are only two of numerous coercive techniques employed. In successful revolutions the emerging new paradigm wins out—often because the older defenders of the previously dominant paradigm retire or die. Its basic resources get fleshed out and extended, and a new period of normal science ensues, now under the direction of the new paradigm.

This narrative is familiar, easy to recite, and since Kuhn’s first book has been applied almost algorithmically to numerous cases from science’s history. Expectedly, it guides much thinking when new scientific revolutions are heralded. Everybody knows what to look for: components of the dominant paradigm, troublesome anomalies, any patterns across them which suggest initial sketches of the revolutionary paradigm, and the various extra-rational persuasive techniques that characterize the revolutionary stage.

In light of its continuing influence, this article’s principal philosophical theme is that, concerning neuroscience over the past 60 years, Kuhn’s model is wrong. There have been revolutions in its fields, and new ones are brewing. But these episodes do not adhere to Kuhn’s model. When it comes to one of science’s hottest interdisciplinary endeavors, real revolutions turn on a different dynamic. The failure of Kuhn’s model is also not due to neuroscience’s languishing in some “pre-paradigm” phase. It is easy to cast current mainstream neuroscience in Kuhnian terms. The basic resources are there.^1^ The problem is deeper. Kuhn’s famous account misses the principal hinge.

My metascientific approach in this essay focuses on two recent cases of neuroscientific revolution, both drawn from cellular/molecular neurobiology, and behavioral neuroscience using animal models. These are the fields I know best. However, I will suggest revolutionary cases from other areas of neuroscience, including one from cognitive neuroscience, which I suspect also illustrate the metascientific points I will develop below.

## Revolutions in Neuroscience: The Role of tool Development

Silva et al. (2014) provide a recent foray into revolutionary neuroscience. The authors derive a Framework of types of experiments, and a set of procedures for integrating results across experiments and labs, by analyzing landmark publications from the field of molecular and cellular cognition (MCC). Using rudimentary causal graph theory they then develop *research maps* of published results, show how to derive *causal maps* of pathways between neuroscientific phenomena from them, and demonstrate the potential for automating these procedures. Together these resources provide a huge aid for rational experiment planning in current neuroscience, with the potential “to make scientific revolutionaries of us all” (Silva et al., 2014, p. 177). Landreth and Silva (2013) provide useful solutions to practical questions about implementing the program.

Like Silva et al. (2014) my goal in this article is to answer a metascientific question. By “metascience” I mean the attempt to understand scientific practice and products on scientists’ terms, as unencumbered by philosophical assumptions or presuppositions about what those practices or products “have to be or do” (Bickle, 2003, chapter 1; Bickle, 2008). In short, I am asking questions about science, only here my question is: what kick-starts actual neuroscience revolutions? Of course, this question presupposes another. Which developments constitute neuroscience revolutions—the real “game changers” in the discipline which set the stage for so much novel work that followed? Consider four developments, from the past 60 years:

- single-cell recordings *in vivo*, and the subsequent assignments of “field” properties (sensory, motor, memorial, etc.) to individual neurons based upon their responses;
- manipulating individual protein components of intra- and inter-cellular signaling pathways in genetically engineered mammals (“knock-outs”, “transgenics”), and measuring their effects *in vivo* using well-established behavioral protocols;
- imaging increasingly smaller regions of the *functioning* human brain; and
- most recently, using light stimuli to activate or inhibit specific selected neurons *in vivo*, while the animal engages in behavioral tasks.

Each of these developments was a *bonafide* revolution. The first came to define reductionistic neuroscience from the late 1950’s through the 1980’s. The second enabled manipulation of hypothesized cellular and molecular causal mechanisms with a precision that far outstripped the capacities of stimulating electrodes and pharmacological interventions, and has subsequently become part of the practices across virtually all of behavioral neuroscience. The third now practically defines the field of cognitive neuroscience.^2^ And I’ll have much to say about the revolutionary nature of the fourth below. Each of these revolutions stemmed directly from the development and justification of a new *experiment tool*—at least one novel to neuroscience at its time. The first revolution stemmed from fine-tip, high-impedance conducting microelectrodes; the second from the application of gene targeting techniques to mammals; the third on positron-emission tomography (PET) and less invasively, functional magnetic resonance imaging (fMRI); and the fourth on technologies both for inducing translation and synthesis of microbial opsin genes in mammalian neurons and delivering the light stimulus to the manipulated neurons. In short: understanding *tool development* is the key to understanding real revolutions in actual neuroscience.

Silva et al. (2014) recognize tool development as one of the three basic categories of experiments in MCC, at the most abstract level of their Framework. But they then focus exclusively on the nature of Connection experiments (a second of their three basic categories), which seek evidence for hypothesized causal relations between neuroscientific kinds, because Connection experiments constitute the bulk of the published research in MCC. The authors thus leave a metascientific treatment of Tool Development experiments for future work. But note that their entire Silva et al. (2014) book is an argument for *developing a new tool* for revolutionizing experiment planning in neuroscience, namely, research maps. Their approach is illuminating. When neuroscientists self-consciously seek to revolutionize some aspect of their discipline, their principal strategy is to develop a new tool.

## Case Study #1: Gene Targeting in Mammalian Neurons

As a first step toward developing a metascience of revolutionary tool development in neuroscience, I examine two detailed case studies, both drawn from the four examples I noted above. I will derive two concepts from investigating these cases: *motivating problem* and *hook experiments*. Silva et al. (2014, chapter 3) coined the term “hook experiment” to denote the first experiments and publications which gained the field of MCC its initial notice. I am building on that usage, although I will have more to say about some specific features of hook experiments. These two concepts hardly exhaust a metascientific analysis of tool development in neuroscience, but they get an analysis started—and distance the subsequent account of revolution in neuroscience immediately from Kuhn’s model.

Consider first the application of *gene targeting technology* into mammalian neuroscience.^3^ The basic outline of this case study is relatively well known within neurobiology (although some of the details I’ll stress often get ignored). This is an important reason why I start a metasceintific analysis of tool development with this case: an unfamiliar project is often best illustrated initially by working with a well-known case. The Benzer lab at Caltech had begun manipulating specific genes in flies, and tracking their behavioral effects, as early as the late-1960’s; by the late-1980’s they and other labs had developed over 20 specific learning and memory fly mutants (with expressive names such as “rutabaga” and “dunce”). Might it be possible to extend such work to mammals, with their far richer behavioral repertoires? This possibility seemed intuitively remote, due to the myriad complexity between gene expression and protein synthesis, and behavior in mammals (as compared to flies); but was enhanced by the work of Capecchi at the University of Utah, who first developed mammalian gene “knock-out” technologies in the mid-1980’s. Thomas and Capecchi’s (1987) lab targeted developmental genes in the mouse, but he asserted confidently that the technique should be applicable to any cloned gene. This work eventually won Capecchi a share of the 2007 Nobel Prize for Physiology or Medicine, shared with co-contributors Martin Evans and Oliver Smithies. The press release for the Prize heralded their work as “the beginning of a new era in genetics” (http://www.nobelprize.org/nobel_prizes/medicine/laureates/2007/press.html).

By the late 1980’s long-term potentiation (LTP), a form of activity-dependent synaptic plasticity (enhancement), provided a beguiling hypothesized neurobiological mechanism for learning and memory. Originally discovered as a physiology laboratory oddity in the 1960’s, the authors of its first systematic experimental investigation speculated explicitly about its potential role in memory (Bliss and Lømo, 1973). Much work, both slice-physiological and behavioral *in vivo*, followed quickly. The circumstantial case for the LTP → memory connection was well known (Lynch, 1986). Then-recently discovered post-synaptic N-methyl-D-aspartate receptors (NMDAR), activated only during periods of strong membrane depolarization (to remove a magnesium ion channel block), which permitted calcium (Ca^++^) and sodium (Na^=^) influx when activated, seemed ideally suited to be part of the mechanism for LTP induction (although the experimentally confirmed molecular details of NMDAR-dependent LTP were then quite sparse). Still, many neuroscientists doubted that the LTP → learning and memory causal connection had been established experimentally, with a reasonable level of scientific confidence.

Morris’s (1989) systematic, multiple-experiment study using D,L-2-amino-5-phosphonopentanoic acid (AP5; also known as APV) was a big step forward. AP5 is a potent and selective NMDAR antagonist, blocking the influx of Ca^++^ into the post-synaptic neuron. Morris (1989) administered AP5 intraventricularly into rats prior to their performing a variety of hippocampus- and non-hippocampus-dependent learning and memory tasks (mostly water maze tasks, known by Morris’s name). In rodent hippocampus slices, AP5 was known to block LTP. Morris (1989) showed that it also decreased memory performance in the hidden platform version of his water maze task, but not the visual platform version. In the hidden platform task, hydrophobic rodents must learn to find a platform submerged below the surface of opaque water in a pool by learning its location relative to numerous distal visual cues on the walls of the room containing the pool. This task is hippocampus-dependent; rodents with bilateral hippocampus lesions are impaired in their ability to learn the hidden platform’s location relative to the visual cues, as compared to sham-lesioned controls. In the visual platform task, a single visual cue at the location of the submerged platform, like a flag on the platform emerging out of the water’s surface, marks the location. This second water maze task is not hippocampus-dependent; although rodents with bilateral hippocampus lesions are slower than sham-lesioned animals to learn the task initially, by the end of standard training periods their performance is statistically identical. These were exactly the pattern of results Morris (1989) obtained with AP5-infused rats.

Intraventricular AP5 infusion also produced motor deficits in the rats, but a careful subsequent experiment suggested that these deficits were independent of water maze performance. Morris (1989) fifth experiment showed that the dosages of AP5 used in the memory experiments were sufficient to block hippocampus LTP *in vivo* without interrupting normal synaptic transmission. Nevertheless, selective pharmacological NMDAR antagonists like AP5 inevitably disrupt synaptic function in subtle ways, potentially interfering with activity in hippocampus circuitry. As Silva et al. (1992b, p. 201) put this concern, perhaps the failure of learning Morris had painstakingly demonstrated “results not from the deficit in LTP, but simply from some other incorrect operation of hippocampus circuits that lack NMDA receptor function”. Obviously, a selective NMDAR antagonist like AP5 could not unravel that potential confound.

Taken together, the predicted general applicability of Capecchi’s gene targeting techniques to mammalian nervous tissue, and the experimental demand to block LTP without disrupting other aspects of synaptic function in order to test the alluring LTP → (rodent spatial) learning and memory hypothesis, constituted the *motivating problem* for the application of gene targeting techniques in mammalian behavioral neuroscience. Other possible solutions to the second feature of this motivating problem seemed reasonable to pursue. One might try to develop more specific pharmacological agents, along with more precise means of their delivery to specific neuronal targets *in vivo*. But this strategy was known to face limits. Even the most selective receptor agonists and antagonists inevitably affect synaptic function; receptor activation is the first (post-synaptic) step of that complex process. Drugs can leak from injection sites into surrounding tissue, particularly in behaving animals, and especially if larger doses are required to elicit desired behavioral effects. Even the most specific drug typically has “off-target” effects. A more promising strategy would seem to be to manipulate individual proteins more directly, in specific intra- or interneuronal molecular pathways underlying quite specific physiological processes. Capecchi’s gene targeting procedure seemed ideal for this task. By “knocking out” the specific gene coding for some judiciously chosen protein product, one could abolish that protein’s specific contribution to the phenomena of interest—LTP, rodent spatial learning and memory, whatever—hopefully without disrupting other aspects of synaptic function, due to the vastly increased specificity of the molecular intervention compared to the best interventions pharmacology could offer. But an immediate question loomed: which neuronal genes/proteins to target, to test experimentally the enticing LTP → (rodent spatial) learning and memory hypothesis?

Like most motivating problems which generate revolutionary tool development in neuroscience, this one was multi-faceted: it was a combination of multiple technical problems with a daunting interactive dynamic.^4^ Beyond the looming question just mentioned lay others. Was Capecchi’s speculation correct, that his gene targeting technique could work for any cloned gene of interest? In particular, could it work for a gene in neurons *in vivo*? Neurons are relatively delicate cells, susceptible to cell death in a variety of ways. The gene target chosen had to code for a protein significant enough in neuronal signaling pathways to block LTP if eliminated, and have downstream consequences all the way to behavior if it was to provide evidence for the LTP → (rodent spatial) learning and memory hypothesis. These effects had to follow from the elimination of a *single gene* and its subsequent protein product. Yet this disruption had also to be specific enough not to disrupt other aspects of synaptic function in excitatory forebrain neurons. Experimenters would have to verify that the targeted gene’s transcription and protein production truly was eliminated. The targeted gene could not interfere with normal development, from the embryonic stem cell stage when the mutation is engineered, up through the adult life stage when the behavioral tests would be administered. Brain development in the mutants had to be normal, from functioning excitatory synapses all the way up to gross anatomy of hippocampus circuitry. The behaving mutants had to possess normal vision, motor capacities, and motivation to solve the behavioral tasks, so the specific mutation had to leave all those phenomena intact. And perhaps most challenging: could all this be accomplished, in one fell swoop?

Notice that the centrality of this first metascientific concept of motivating problem for a new tool’s development introduces distinctively un-Kuhnian aspects to the subsequent account of neuroscience revolutions. First, experimental tools are just one component of Kuhn’s multi-component concept of a paradigm; my claim here is that this one component is central to actual neuroscience revolutions. But notice that a new tool’s motivating problem is even more specific: on a specific tool developed for a specific experimental purpose. Second, a revolutionary new tool’s motivating problem is not a Kuhnian anomaly. In the case at hand all serious practitioners in the field recognized the limits of the best existing pharmacological tool (AP5) put to use to achieve the overarching experimental goal of testing directly the LTP → (rodent spatial) learning and memory hypothesis. It was widely accepted that the existing tools failed to achieve this specific experimental end; further applications of the existing tools were not expected to succeed beyond what Morris (1989) had accomplished. Even the most methodologically conservative experimentalists recognized the need to develop some new tool—at a minimum, some new pharmaceutical compound other than a post-synaptic receptor agonist or antagonist, and a more specific way to deliver it *in vivo*. This is not to say that no further experimental work with compounds like AP5 would be done. It still had numerous experimental uses. But its use alone could not further confirm the hypothesized LTP → (rodent spatial) learning and memory causal connection; and no one was puzzled about why.^5^

The combination of features of a potentially revolutionary new tool’s motivating problem illustrated in this case study also explains why its initially successful *hook experiments* are so surprising to the research community. Even scientists “in the know” about early attempts to develop the new tool assign a low probability to these experiments succeeding. Due to the subsequent reaction to these experiments’ publication, not to mention the number of citations which follow quickly and sustain, there is usually not much controversy over which experiments constitute a revolutionary new tool’s initial “hooks.” They are the first published results using the tool: (1) typically in a top journal in the field; which (2) specifically apply the tool to the targeted population of experimental interest; and (3) address a phenomenon in the targeted field of inquiry. For gene targeting techniques in mammalian behavioral neuroscience, these initial hook experiments were published in Silva et al. (1992a,b) and Grant et al. (1992). All three articles were published in *Science* (condition 1), all targeted a specific gene/protein in mice (condition 2), and all tested effects of the genetic intervention on LTP in hippocampus slices using then-state-of-the-art electrophysiological techniques and measures, and long-term learning and memory in water maze and other hippocampus-dependent (declarative, explicit) tasks (condition 3).

Alcino Silva, working in Susumu Tonegawa’s lab, used Capecchi’s gene targeting technique to knock out the gene for the α-isoform of the calcium-calmodulin-dependent kinase II α-CaMKII). Some features of this protein were then known. It is highly enriched in the post-synaptic densities of mammalian forebrain excitatory neurons, including hippocampus and neocortex. It plays a role in NMDAR-dependent LTP. It is activated by calmodulin loaded with intracellular Ca^++^, whose influx into the post-synaptic cell is through activated NMDARs. Activated α–CaMKII phosphorylates numerous other post-synaptic proteins, thereby activating them, and itself remains activated via autophosphorylation after Ca^++^ influx ceases. In these ways α-CaMKII already met some key requirements of a computational model of a molecular mechanism to strengthen synapses developed a few years earlier by Lisman (1985) and Lisman and Goldring (1988). Lisman’s model itself was a more specific version of Hebb (1949) famous “fire together-wire together” speculation.

Silva et al. (1992b) constructed the plasmid that disrupted the *α-CaMKII* sequence, transfected the plasmid into mice embryonic stem cells, injected the stem cells into blastocytes, inserted the blastocytes into pseudo-pregnant females, bred the resulting chimeric males with wild-type females, and after multiple crosses confirmed the expected Mendelian ratios (wild-type homozygous, wild type-mutation heterozygous, mutation homozygous) for a non-lethal mutation. Homozygous α-CaMKII mutants completely lacked α-CaMKII messenger RNA (mRNA) and protein in forebrain tissue, or any truncated form of them, but showed normal mRNA and forebrain protein levels for the closely related β-CaMKII isoform. Coronal sections through hippocampus revealed no gross anatomical abnormalities in cell types, distributions, or axonal pathways. Aside from “increased jumpiness” or “nervousness” when handled by humans, mutant mice behaved normally. The mutation had no effect on long-term survival under standard laboratory housing. In light of all these preserved features, wild-type littermates could be used as controls for experimental mutant mice, for both slice-physiological and behavioral studies.

Electrophysiological studies using hippocampus slices found no differences between α-CaMKII mutants and controls in synaptic currents, in either NMDA or non-NMDA components. Measures included peak size, time course, and ratio of NMDA to non-NMDA components. Nor were there any differences in the dependency of NMDAR channel conductance on neuronal membrane voltage potentials (Silva et al., 1992b, Figures 3, 4). NMDAR function in α-CaMKII mutant mice slices was normal. So this initial hook experiment delivered successfully on one key desideratum of the motivating problem: the targeted mutation did not disrupt synaptic function. The key confound plaguing the most careful and specific pharmacological studies of the LTP → (rodent spatial) learning and memory connection was resolved. But that part of the motivating problem was, relatively speaking, the easy part. Could this single targeted gene mutation produce the needed physiological and behavioral effects?

Silva et al. (1992b) next investigated the second desideratum, impaired LTP in hippocampus slices, using both field potential recordings to survey populations of hippocampus neurons and more sensitive whole-cell recordings. They demonstrated deficient LTP in mutant hippocampus slices. Slices from littermate controls showed normal tetanus-driven LTP for all time periods measured (up to 1 h after tetanus). Aside from a brief post-tetanus stimulus potentiation (about 1 min), synaptic strength in mutant slices was unchanged from baseline levels, and remained so even after a second tetanus with increased pulse trains was delivered. In whole-cell recordings most all hippocampus neurons in control slices exhibited normal LTP, while only a small fraction of α-CaMKII neurons did. Quantal analysis did reveal that the LTP induced in the small fraction of α-CaMKII neurons displaying it was normal (Silva et al., 1992b, Figures 6–8, and Table 1). So this single gene/protein mutation reliably diminished LTP in hippocampus neurons.

Silva et al. (1992a) investigated the third desideratum on the motivating problem, behavior *in vivo* in rodent hippocampus- and non-hippocampus-dependent learning and memory tasks. α-CaMKII mutants were slower to learn the non-hippocampus-dependent visible platform version of the Morris water maze task initially, but over a standard 2-day, 12-trial training period quickly matched control performance. Interestingly, this pattern mimicked both hippocampus-lesioned and AP5-administred animals (discussed above). On the hippocampus-dependent hidden-platform version, α-CaMKII mutants never learned to locate and mount the platform as quickly as controls, over either a standard 3-day or 5-day training period. In probe trials after training, where the platform was removed, mutants spent significantly less time in the maze quadrant where the platform had been located during training, and crossed the platform’s training location significantly fewer times, than did littermate controls. They also crossed the training location of the maze significantly fewer times compared to controls in a small number of random-platform trials interspersed during training, where the hidden platform was changed to a new location (Silva et al., 1992a, Figures 1–4). However, α-CaMKII mutants performed normally compared to controls on a water-filled plus maze task, which requires animals to use a single distal visual cue to learn which arm holds the hidden platform, (in contrast with the spatial relations between platform location and numerous distal visual cues, required on the hidden-platform Morris water maze task; Silva et al., 1992a, Figure 5). Thus the mutants’ failures to learn the hidden-platform Morris water maze task were not due to an inability to see the distal cues, or to learn an association between escape and the distal visual environment. Interestingly, α-CaMKII mutants also demonstrated other subtle behaviors similar to mice with hippocampus lesions (in addition to the “jumpiness” to human contact noted above), such as increased exploration and activity in open fields and enclosed Y-mazes.

Silva et al.’s (1992a) own words about their results illuminate the dual metascientific notions of motivating problem and initial hook experiments developed here. They assert that their work “strengthens considerably the contention that the synaptic changes exhibited in LTP are the basis for spatial memory.” Perhaps more surprisingly, and for the first time, it “demonstrates that a mutation in a known gene is linked to a specific mammalian learning deficit, and indicates that single genetic changes can have a selective but drastic impact on learning and memory.” Finally, and with an eye to the future use of the tool they had demonstrated as applicable to mammalian behavioral neuroscience, they predict that “other similarly constructed mice with mutations in judiciously chosen genes will be useful for studying mammalian behavior” (Silva et al., 1992a, p. 210).

Learning and memory neurobiologists did not have to wait long for this prediction to be met. Five months later another set of initial hook experiments was published. Grant et al. (1992) working in Eric Kandel’s lab, engineered mutations in mice to the genes for each of the four nonreceptor tyrosine kinases, *src*, *fyn*, *yes*, and *abl*. The motivating problem for Grant et al. (1992) work was that for Silva and collaborators: the widely accepted limits of pharmacological agents and their method of application adequately to test the LTP → (rodent spatial) learning and memory connection. Tyrosine kinase inhibitors had been shown to block the induction of LTP without affecting normal synaptic transmission, but the existing drugs lacked the specificity necessary to identify the specific tyrosine kinases involved. Grant et al. (1992) used Capecchi’s gene targeting technique to knock out each of these four nonreceptor tyrosine kinases in different mouse mutants. Synaptic transmission in hippocampus slices encompassing CA3-to-CA1 circuitry from each of the four mutants was normal compared with littermate controls, including maximum field EPSPs and paired-pulse facilitation (Grant et al., 1992, Figure 2). LTP in CA1 neurons was normal in *src*, *yes*, and *abl* mutants, but impaired in *fyn* mutants, in both field EPSP and population spike. Strong intensity tetanus stimulation produced a reduced form of LTP in *fyn* mutants, and was blocked by applications of AP5. Whole-cell patch clamp recordings indicated that the NMDA component of excitatory currents in the *fyn* mutants was normal; synaptic depolarization of the post-synaptic cells produced by the input was adequate to activate NMDAR function (Grant et al., 1992, Figures 4, 5). Behaviorally, just like the α-CaMKII mutants (and hippocampus-lesioned and AP5-administered rodents), *fyn* mutants were initially slower to learn the visual-platform version of the Morris water maze task, but eventually matched control performance during standard training durations. However, they never matched control performance on the hidden-platform version, either during standard (7-day) training, or on probe trials after training on either time in target quadrant or number of target crossings (Grant et al., 1992, Figure 6). LTP and behavior in hippocampus-dependent spatial learning both were normal in *src*, *yes*, and *abl* mutants. However, *fyn* mutants displayed a developmental deficit, in the arrangements of hippocampus dentate gyrus granule neurons and their target CA3 pyramidal neurons (Grant et al., 1992, Figure 7).

These authors were likewise enthusiastic about the general applicability of this new gene targeting tool, now demonstrably feasible for mammalian behavioral neuroscience by work from two labs: “In addition to their role in the study of behavior and learning, targeted disruption of genes provides a powerful tool for examining the role of specific proteins in the function of the brain” (Grant et al., 1992, p. 1908). And these initial hook experiments stuck. In October 1992, 4 months after the Silva et al. (1992a,b) articles had been published and 2 months before the Grant et al. (1992) article appeared, Morris himself, with Mary Kennedy, published a review, “The Pierian Spring” in *Current Biology*, with the subtitle “mutant mice engineered to lack an enzyme critical for long-term synaptic plasticity are deficient in spatial learning” (Morris and Kennedy, 1992, p. 511). They outlined the genetic engineering procedures used, and the electrophysiological and behavioral investigations employed, for non-specialists, and closed with a section on “Implications and potential.” The α-CaMKII knock-out was “an ingenious piece of molecular engineering”; the LTP and behavioral deficits “were by no means a foregone conclusion”; the work “should be recognized as the considerable achievement it truly represents.” The findings “vindicate and extend earlier results” (namely, Morris’s own!) (Morris and Kennedy, 1992, p. 513). The authors note that this work was not without its own problems. The learning capacities in mutants might have been underestimated. The subtlety of the effects of eliminating so significant a post-synaptic protein in forebrain excitatory neurons might reflect compensatory effects of other CaMKII isoforms. α-CaMKII was eliminated completely from the mutants’ brains, including from all regions and pre-synaptically, and so these specific mutants could not be used to address the then-still raging controversy over whether LTP was mediated pre-synaptically, post-synaptically, or both. α-CaMKII is also prominent in neocortical excitatory neurons, and consolidation and long-term storage of spatial memories probably occurs there, in addition to hippocampus; that phase of memory induction may too depend on α-CaMKII activation, which would also be blocked in the mutants. Despite these problems, however, these first hook experiments were “an auspicious beginning and likely to fund a small industrial revolution.” Rather than treading lightly with this new tool, neuroscientists interested in the brain mechanisms of learning and memory “should, as Pope went on to write, “Drink deep, or taste not the Pierian Spring”^6^ Morris and Kennedy, (1992, p. 514).

Neuroscientists, and not just those working on learning and memory, certainly heeded Morris and Kennedy (1992) recommendation. Gene targeting quickly became standard practice across neurobiology, which greatly expanded the then-nascent search for molecular mechanisms of “higher” functions. This new experimental tool brought molecular neuroscience to the prominence in the discipline it maintains to the present day. But the initial hook experiments which expose a new tool to the purview of professional scientists are just one component of genuinely revolutionary tool development. A second kind of hook experiment garners the tool even wider appeal and application. Identifying these “second-phase” hook experiments for any given revolutionary tool is more controversial, as there are often a number of viable candidates. For gene targeting techniques in behavioral neuroscience, however, the general shape of these second-phase hook experiments was anticipated by both Silva et al. (1992a) and Grant et al. (1992): engineered genetic mutations more specific than whole-gene knock-outs. Silva et al. (1992a, p. 210) write: “Perhaps even more useful would be … mice with mutations directed to specific regions of the brain. Construction of such mutant mice may be feasible”. Grant et al. (1992, p. 1908): “To strengthen the links between Fyn, hippocampus LTP, an spatial learning, it will also be necessary to specifically manipulate the expression of mutant forms of Fyn, restricted only to the hippocampus”. The continued development and use of gene targeting in mammalian behavioral neuroscience quickly exceeded both groups’ expectations. Not only did the regional specificity both envisioned follow quickly, but so did temporal spexcificity, the capacity to express engineered gene mutations only during specific developmental phases, e.g., in adult rodents, and even at specific times during behavioral protocols.

A new molecular target for learning and memory studies emerged first. Günther Schütz’s developmental biology lab developed a homozygous knock-out mouse deleting the gene for the α- and δ-isoforms of cAMP-response element-binding protein (CREB). CREB is a transcriptional enhancer prominent in many tissues. Earlier work with flies, including gene targeting work, and with the sea slug *Aplysia californica*, suggested that CREB, when activated via phosphorylation, enhanced expression and synthesis of a variety of genes and proteins important for LTP (or for “long-term facilitation,” as the process is called in invertebrates) and for invertebrate forms of associative learning. Working with Schütz’s *CREB* mutants in Silva’s lab at Cold Spring Harbor, Bourtchuladze et al. (1994) showed they were deficient in long-term memory tasks (24-h delay), but intact in learning and short-term memory (30–60 min delay) on these same tasks. Behaviors included both hippocampus-dependent (single-training trial aversive contextual fear conditioning, where the animal is conditioned with a foot shock after initial exposure to a new environment, and the hidden-platform version of the Morris water maze) and non-hippocampus-dependent tasks (single-training trial cued Pavlovian aversive conditioning to an auditory tone). These results suggested that CREB plays a key role in the consolidation of memory from short-term to long-term form. Hippocampus slice-physiology work proved consistent with this interpretation of the behavioral results. LTP in mutant slices was smaller than littermate controls and declined to baseline by 90 min (Bourtchuladze et al., 1994).

CREB mutants immediately became the target of extensive behavioral neuroscience investigations, and the same pattern of results emerged for an large variety of rodent memory tasks: intact short-term performance but impaired long-term performance on the same tasks. This pattern held both for standard memory consolidation and for reconsolidation after stimulus re-presentation. The importance that CREB mutants played for learning and memory research over the next few years after Bourtchuladze et al.’s (1994) second-phase gene targeting hook experiments is nicely summarized by Dudai (2002, p. 65) in his introductory sourcebook for memory research:

CREB is one of the most commonly used acronyms in neurobiology these days, and also one of the few words in the jargon of molecular biology that even experimental psychologists and computational neuroscientists might have encountered. And if they didn’t, they should. Because the more we advance our knowledge in molecular biology the more we realize that CREB plays a pivotal role in the response of neurons to external stimuli.

Dudai’s remarks demonstrate one crucial feature of second-phase hook experiments for genuinely revolutionary tools: widespread dissemination of the tool and its results to a wider public, beyond the specialists working in the field that developed it.^7^

Neuroscientists were also soon attracted to a different kind of gene engineering technique, the transgenic approach. This approach involves inserting an extra copy or copies of a cloned gene into the DNA of mammal embryonic stem cells, often attached to a promoter region which limits expression to specific neurons. Every cell in the mutants’ bodies contains the extra transgene copy or copies; but transgene transcription (and subsequent protein synthesis) only occurs in those tissues or specific cells possessing the promoter molecule in sufficient abundance. An early influential use of this approach in behavioral neuroscience was Abel et al. (1997) in Kandel’s lab. They inserted extra copies of the gene for regulatory subunits of the cAMP-dependent protein kinase A (PKA) molecule into mouse embryonic stem cells, with a CaMKII promoter which limited transgene expression to forebrain regions, including hippocampus (but excluding significant expression in amygdala). PKA plays a prominent role in the cAMP-PKA-CREB intracellular signaling pathway. In mammalian post-synaptic neurons, increased intracellular cAMP (driven by activation of dopaminergic modulatory neurons) binds to regulatory submits of PKA molecules. This binding frees catalytic PKA subunits to translocate to the neuron’s nucleus and phosphorylate CREB molecules. Phosphorylated CREB in turn drives gene expression and synthesis for both regulatory and effector proteins which restructure active synapses, leading to increased EPSPs to subsequent glutamate pre-synaptic release. In Abel et al. (1997) R transgene mutants, the extra R PKA (regulatory) subunits available in neurons in which the transgene is expressed quickly bind up PKA catalytic subunits freed by increased cAMP, blocking that early step in the cAMP-PKA-CREB pathway. At the time that these second-phase gene targeting hook experiments were carried out, CREB’s role in LTP and memory consolidation had already been established, with Bourtchuladze et al.’s (1994) CREB^−^ mutants (see above). But CREB is phosphorylated through numerous intracellular signaling pathways, and the CREB knock-out mice couldn’t distinguish between which of these pathways was crucial for its role in late-phase LTP and memory consolidation. Attempts to knock out the PKA gene using Capecchi-style gene targeting techniques had been inconclusive.

Abel et al.’s (1997) founder mutant mice bred successfully and transmitted the transgene to offspring. The promoter limited significant transgene expression to forebrain areas, including all regions of the hippocampus, but hippocampus gross anatomy was otherwise unaffected. Hippocampus PKA activity was reduced in R transgenic mice, but basal synaptic transmission, paired-pulse facilitation, and post-tetanus potentiation to one- or two-train tetanus stimuli were all unaffected in mutant hippocampus Schaffer collateral (CA3→CA1) pathways. However, late-phase (L-) LTP was reduced significantly to four-train stimuli in mutant slices for all time periods tested (starting at about 40 min and measured up to 180 min post-tetanus). Behaviorally, while escape latency during training on the hidden-platform version of the Morris water maze task in R transgenic mutants did not differ from littermate control performance, mutants were deficient in both time-in-target quadrant and number of target crosses in probe trails administered after training (Abel et al., 1997, Figure 5). More importantly, R transgenic mutants were intact on short-term (1-h delay) contextual fear conditioning, significantly impaired on the long-term (24-h delay) version, but unimpaired on both short-term and long-term versions of tone-foot shock (Pavlovian) conditioning compared to control mice. The latter task is amygdala-dependent, where the R transgene was not expressed significantly due to the CaMKII promoter.

Two more recent second-phase hook experiments bring us up to date on the development of gene targeting targeting techniques in behavioral neuroscience. The first used conditional gene knock-outs, which temporarily and reversibly repress a targeted gene’s expression and protein synthesis. An important hook experiment for this development was Kida et al. (2002), who fused a CREB α-isoform repressor (IR, with an alanine residue substituted for the serine residue at positon 133) to a ligand-binding domain of a human estrogen receptor mutated to become activated by the drug tamoxifen (TAM). Kida et al. (2002) derived transgenic mice expressing this CREB^IR^ mutation under the control of an α-CaMKII promoter to render it active only in excitatory forebrain neurons. The CREB^IR^ isoform protein competes with phosphorylated CREB for binding sites on target regulatory and effector genes, and represses expression at those sites, but only for the duration of systematic TAM administration. At all other times the CREB^IR^ transgene is silent with no effect on CREB activity (including throughout brain development). CREB^IR^ activation did not disrupt short-term memory (2-h delay) for (hippocampus-dependent) contextual fear conditioning, but disrupted its consolidation into long-term form (24-h delay). CREB^IR^ activation also impaired the stability of reactivated fear conditioned memories in mutants, Mice were returned to the context paired with the foot shock 24 h after initial training (the “reactivation” phase) and were then retested in the context 24 h later. Transgenics whose CREB^IR^ repressor isoform was activated by TAM during memory reactivation froze significantly less to the context at retest than did the three control groups: wild-type littermate controls, transgenics whose CREB^IR^ repressor was not activated, and transgenics whose CREB^IR^ was reactivated but whose context-fear memories were not reactivated by context re-presentation. The results from this second-phase gene targeting hook experiment with a temporally conditional knock-out mutation provided a crucial piece of evidence that CREB is part of the molecular mechanism for both consolidation and reconsolidation of hippocampus-dependent (“declarative”) memory.

The use of engineered virus vectors to deliver transgenes via the usual process of the virus replication cycle also enabled scientists to bypass possible developmental abnormalities which result from insertions in embryonic stem cells, and to insert genes of interest directly into the neurons of adult mammals. Delivery of virus vectors via microinjection further limited infection, and hence transgene transfer, to specific neural regions. An early application of this technique to behavioral neuroscience was Josselyn et al.’s (2001) work, in Michael Davis’s lab. Rodents show increased L-LTP, and increased long-term learning, in tasks requiring multiple training sessions when training sessions are *spaced*, with non-training intervals in between, rather than when *massed* into a single multiple-exposure session. Josselyn et al. (2001) showed that rats who received infusion of a transcription enhancer isoform of the *CREB* gene, with the virus vector microinjected bilaterally into the basolateral nucleus complex of the amygdala, showed significantly enhanced long-term memory with massed training on a fear-potentiated startle paradigm involving four light-shock pairings. No control groups showed any increase in long-term memory performance to massed training: rats microinjected with a control substance, rats microinjected with a mutated inactive form of the CREB gene, rats microinjected with a Lac-Z gene with no learning and memory effects, rats whose microinjections had missed or whose infections had extended beyond the amygdala basolateral nucleus, and rats microinjected with the CREB gene directly into the caudate nucleus. There were no differences across groups in immediate reactions to shocks, explicitly unpaired training failed to produce long-term memory in the CREB-boosted rats, and increased long-term memory was retained in CREB-boosted rates 14 days after massed training. As Josselyn et al. (2001, p. 2410) remark, their results were first to show that “overexpression of CREB in a specific mammalian brain region at a specific time enhances LTM [long-term memory]”. These types of experiments, labeled “mimicry” by Sweatt (2009) and “positive manipulations” by Silva et al. (2014), provide a kind of evidence widely recognized as necessary for scientific confidence that a hypothesized causal connection, e.g., CREB expression in specific brain regions → enhanced LTM, has genuinely been established.

The gene targeting revolution across mainstream neuroscience is now fully accomplished. The technique is a mainstay of work in cellular, molecular, and behavioral neuroscience across all phenomena. Any research institutions of any serious standing now has a core facility to develop conditional knock-out and transgenic rodents (and other mammals), not just for neurobiologists, but for all fields of bio-medical inquiry. The vast increase in our knowledge of direct cellular and molecular mechanisms for “higher-level” biological phenomena of all types can be traced to the application of these techniques. To deny this scientific development the status of a revolution would be simply to misunderstand its significance in current scientific inquiry. But the actual dynamics of this revolution, in recent neuroscience as in all other fields, hinged on *the development of this tool*, especially the roles of its motivating problem, and initial and second-phase hook experiments. These features, rather than anything deemed crucial on Kuhn’s famous model, were this scientific revolution’s vanguard.

## Case Study #2: Optogenetics and Designer Receptors Exclusively Activated by Designer Drugs (DREADDs)

For a second detailed case study of an actual neuroscientific revolution, consider *optogenetics*, an experimental technique in existence in neuroscience for scarcely one decade (even though some of its resources have been standard in molecular biology for three times that long). This is an especially interesting case of scientific revolution, not only because the science itself is so intriguing and was initially so implausible, but also because it is a revolution still in the making. Yet it is difficult to deny that optogenetics’ impact on neuroscience has already been revolutionary. Since it was named “Method of the Year” in 2010 by *Nature*, the number of optogenetics publications searchable annually has increased from roughly 100 to already close to 900, through only the first half of 2015 (Deisseroth, 2015). Recent reviews discuss experimental results in mammalian behavioral neuroscience ranging from learning and memory, sleep/wake transition, addiction, motivation, reward, social interactions, anxiety, and models of neurological disease and trauma.^8^

Being an ongoing, still potential revolution implies, of course, that the optogenetics revolution might not come to fruition. Otchy et al. (2015) report results which challenge an assumption behind the experimental use of rapid and reversal manipulations of neural activity like optogenetics, that the observed behavioral effects reflect the function of the manipulated circuits. This assumption is problematic because of indirect effects of the manipulation on independent downstream circuits, which are difficult to control. So noted. Despite its role in kick-starting neurobiological revolutions, tool development is just one aspect of progressing science, revolutionary or otherwise.^9^

One feature that makes optogenetics appealing as an experimental tool is the unprecedented control it offers over the activation (or inhibition) of specific targeted neurons in living, behaving animals. Various classes of light-responsive proteins, most prominently the microbial opsins from algae, serve as membrane-spanning channels for cation (e.g., sodium, Na^+^) or anion (e.g., chloride, Cl^−^) influx, thereby depolarizing or hyperpolarizing the cell when activated. The DNA coding for these opsins is removed and engineered to be expressed effectively in mammalian DNA. It is inserted via a virus vector, nowadays typically an engineered adeno-associated virus whose own genetic material has been removed to allow insertion of the engineered opsin DNA. The virus vector is then micro-injected directly into specific regions of mammalian brains, typically mice or rats. Specific neurons infected by the virus take up the engineered opsin DNA by the usual mechanisms of the viral replication cycle. They are thus poised to begin expressing the engineered gene and synthesizing the new protein, under the control of the promoter region of the engineered gene. These synthesized opsin proteins embed in the infected neurons’ membranes. They now can be activated by a light stimulus to pass the specific cation or anion into these neurons, thereby activating or inactivating them at the experimenters’ command—by switching on the laser or light-emitting diode (LED) light source embedded in the animal’s brain. The stimulating light source is inserted through chronically implanted cannulae cemented permanently into the animals’ skulls. Activating (or inactivating) specific neurons can have downstream effects of activating (or inactivating) entire neuronal circuities. Typically the engineered DNA for an imaging protein (mCherry, green florescent protein, yellow fluorescent protein) is also coupled to the engineered DNA for the opsin protein in the virus vector. All neurons infected by the virus will also express the imaging protein, and glow a particular color (red, green, yellow) in brain tissue slices under standard light microscopy. Experimenters can thus verify exactly which neurons expressed the opsin protein, and were activated (or inhibited) by the light stimulus (this is all now standard procedures for the use of viral vectors in gene targeting, introduced toward the end of the previous section).

Those are the basics. Further refinements to these techniques typically depend upon the specific experimental questions optogenetics is used to address. For example, memory researchers often insert the opsin transgene against an already-genetically engineered rodent background that activates the expressed opsin transgene only in infected neurons that are also highly activated by the target memory stimulus. Many types of highly active neurons express c-fos, the protein product of an immediate early gene, which in turn can drive the production of an engineered tetracycline-responsive element promoter protein, which turns on opsin transgene expression only in infected neurons which are also the most highly active. At all other times this background tetracycline-transactivator system is kept silent by doxycycline in the animals’ diets. This trick enables experimenters later to turn on via the light stimulus all and only those infected neurons which were most active during memory formation—the hypothesized cellular engram for the memory stimulus.

Designer receptors exclusively activated by designer drugs (DREADDs) technology is a more recent refinement which dispenses with optogenetics’ inserted light stimulus. Instead of an engineered microbial opsin gene, the gene for an entire protein receptor for a designer drug—a compound, such as clozapine-N-oxide (CNO), which nowhere occurs naturally in biological tissue—is inserted via the virus vector. These engineered receptors are metabotropic (coupled to a G-protein), to induce either bursting (G_q_-type protein) or silencing (G_i_-type protein) in infected neurons. The designer drug is then administered systemically, either through peritoneal injections or diet. It binds to the engineered receptors in the infected neurons, activates the attached G-protein complex, and induces bursting or silencing in those neurons for the extent of the pharmacological activity of the designer drug.

Neurobiologists are now furiously exploring the relative advantages and disadvantages of optogenetics vs. DREADDs. The lack of need for an implanted light source is one technological advantage of DREADDs. So can be the number of neurons activated by the drug. Systemic administration of the designer drug means that all the neurons infected with and expressing the transgene for the designer receptor will be affected, no matter where they are located in the brain. In optogenetics, only those neurons infected, expressed, and in the range of the stimulating light source are affected. This extent of the drug’s effect is a two-edged sword, however. It puts a premium on micro-injection placement of the DREADD-containing virus and subsequent verification of this placement. Leakage of the virus into other non-target neurons, and subsequent expression of the designer receptor in them, can confound behavioral results. On the other hand, DREADDs’ temporal control is tied to the pharmacokinetics and -dynamics of the designer drug—its absorption, distribution, metabolism into inactive metabolites, and excretion. This slow and imprecise temporal effect contrasts sharply with optogenetics’ temporally much more tightly controlled on/off light switch. Both techniques permit anterograde-retrograde interactions, in which the specific neuronal targets of the axons of the optogenetics- and DREADD-activated neurons can be identified and in turn manipulated.

The two metascientific concepts illustrated in the case of revolutionary gene targeting techniques in neuroscience are also present in the ongoing optogenetics revolution. The motivating problem for optogenetics stemmed from the causal-mechanistic explanatory goals of mainstream neurobiology. To test hypotheses about the causal role played by specific neurons to produce behaviors routinely taken as indicators of particular cognitive functions in mammalian models, experimenters need the capacity reliably to intervene into the hypothesized mechanisms *in vivo*—to activate or inhibit them in the behaving organism, as directly and as efficiently as possible. They can then measure the effects of these experimental interventions on the behaviors of the organism, in tightly controlled experimental protocols routinely taken to be indicators of the target phenomenon. This task has become more difficult in light of continued discoveries of the sometimes sparse anatomical distributions of the hypothesized cellular mechanisms. Pharmacological interventions easily can be confounded by the spread of the drug to nearby non-target neurons; nature doesn’t always cooperate to clump target neurons together into discrete cortical columns or microcolumns to make stimulation by microelectrodes feasible. The intriguing suggestion of using light stimuli to activate or inhibit target neurons was already on offer (Crick, 1999). The “obvious” solution was to develop some way to induce the distributed specific target neurons themselves to synthesize light-sensitive ion channels. But that solution explodes quickly. For speed of activation or inhibition the expressed ion channels should be ionotropic, which simply open direct channels for a specific cation or anion influx or efflux. Engineered gene expression and protein synthesis would have to be at levels both safe and in sufficient numbers to have enduring effects on neuron membrane potentials. The activating light stimulus had to be deliverable to induced neurons safely, often deep in the brain, yet with sufficient strength to induce sufficient activity in enough of the target neurons to affect circuit activity. And all of this had to be implemented *in vivo*. But if successful, neurobiology would then have a great new experimental interventionist tool to explore causal-mechanistic hypotheses relating activity in specific neurons to behaviors—including behaviors routinely taken to indicate the occurrence of particular cognitive functions.

This was optogenetics’ motivating problem. Like most motivating problems which generate revolutionary tool development in neuroscience, including the example of gene targeting techniques discussed above, this was a composite of many technical problems with a daunting interactive dynamic.^10^ Deisseroth (2015), in whose lab optogenetics in its current form was first successfully developed, summarizes this situation nicely. The three core features of the optogenetics technique include the engineered microbial opsin genes and proteins; general methods for targeting opsin expression to well-defined neurons; and methods for guiding light stimuli to brain regions, often deep below the cortical surface, while the organism carries out the behaviors of interest. Each component problem individually faced serious difficulties; but together they constituted “a biological three body problem in which it was hard to resolve (or, even more importantly, to motivate attempts to resolve) any one of the three challenges without first addressing the other components” Deisseroth (2015, p. 1214). Ion currents through microbial rhodopsin proteins were predicted to be very small; hence high gene expression and light-intensity levels seemed necessary. These levels of expression and synthesis had to be sustainable in neurons, biological cells well known to be detrimentally sensitive to membrane protein overexpression, and to side effects of heat and light. All this plus the specificity of expression had to be achieved while minimizing cell toxicity, in order to be applicable *in vivo*. Furthermore, attempts were already underway to use engineered metazoan rhodopsin genes and channel proteins (which mediate photon transduction in vertebrate retina), which reduced initial enthusiasm for trying to solve these technical problems using a family of genes so foreign to the mammalian genone.

Again, this combination of features of the motivating problem for a revolutionary tool development illustrates why the successful initial hook experiments were so surprising. Even scientists “in the know” about early attempts to develop optogenetics in the form it took assigned a low probability to these experiments succeeding. To repeat from above, thee experiments are the first published experiments using the technique: (1) usually in a top journal in the field; (2) applying it to the targeted experimental population of research interest; while (3) addressing a phenomenon in the target field of inquiry. For optogenetics, this was the Adamantidis et al. (2007) study from the Deisseroth lab. Condition (1) was met: the article was published in Letters to *Nature*.

Adamantidis et al. (2007) engineered a lentivirus using the promoter region of the mouse (condition 2) prepro-hypocretin gene (*Hcrt*). This limited expression of the engineered microbial opsin gene, channelrhodopsin2 (*ChR2*) to hypocretin- (also known as orexin-) producing cells. They micro-injected this virus vector into mouse lateral hypothalamus. Expression persisted and was highly specific to Hcrt-producing lateral hypothalamic neurons. Electrophysiological studies with tissue slices *in vitro* showed that ChR2-expressing Hcrt neurons reliably responded to light stimuli with volleys of action potentials (Adamantidis et al., 2007, Figure 1). Optogenetic photostimulation of ChR2-expressing lateral hypothalamus Hcrt mice also activated infected neurons *in vivo*, as measured by increased *c-fos* expression specifically in these neurons. And optogenetic photostimulation to lateral hypothalamus *in vivo* in ChR2-Hcrt mice outfitted for chronic electroencephalogram (EEG) and electromyogram (EMG) recordings showed that direct activation of these neurons significantly decreased latency to wakeful state, from both slow wave sleep (SWS) and rapid eye movement (REM) sleep (condition 3), as compared to both baseline (non-photostimulated) latency in these same mice and photostimulation in control mice (who received the virus vector injections minus the *ChR2* transgene; Adamantidis et al., 2007, Figure 3). Effects of photostimulation appeared limited to sleep-wake transitions since duration of waking EEG and EMG events did not differ significantly across groups. These effects were dependent upon frequency of the photostimulation, however, as 15 ms light pulses delivered at 1 Hz did not elicit them, while all frequencies greater than 5 Hz did. And the mechanism of these photostimlation-induced effects influenced the sleep-wake circuitry by affecting Hcrt release in the infected neurons. Administration of a single dose of an Hcrt receptor (type 1) antagonist (SB334867) blocked this photostimulation effect on sleep-wake transition latency, from both SW and REM sleep (Adamantidis et al., 2007, Figure 4).

As Deisseroth himself notes, the Adamantidis et al. (2007) study was the first experiment to demonstrate that

it was possible to selectively target a microbial opsin gene with high specificity and penetrance to a defined population of neurons deep in the brain of adult mice … to play in a broad range of spike patterns throughout an optical fiber to these cells, to collect simultaneous multimodal system readouts during freely moving behavior, … and to demonstrate a causal role for defined activity patterns in specific brain cells in natural behavior.

—(Deisseroth, 2015, p. 1216)

Subsequent applications of developments in gene targeting, and opsin genomics and engineering, quickly contributed to the rapid increase in published optogenetics results over the subsequent few years.

As we saw in our first case study, the initial hook experiments which expose a new experimental tool to the purview of a given field’s research specialists are important; but genuinely revolutionary tools garner even wider appeal by way of second-phase hook experiments. As it was for the case of gene targeting techniques, identifying the key second-phase hook experiments is more controversial, as there are a number of viable candidates. In the case of optogenetics, however, arguments for identifying some specific second-phase hook experiments are aided because the technique has penetrated into the awareness of the general (scientifically literate) public. One set of recent optogenetic experiments in particular stands out: work on reactivating the cellular engrams of specific memories, and pairing these reactivations with new memorial information to create false memories in rodents. This work came from Tonegawa’s lab. One behavioral protocol the lab employs is contextual fear conditioning, a hippocampus-dependent (“declarative”) rodent memory task. This is the same behavioral protocol used by Abel et al. (1997) with their R-subunit PKA mutants, discussed in the section above. The rodent is placed in a novel context, a cage with an internal environment it has never encountered previously, allowed to explore for a short duration (1–2 min), and then is foot-shocked. Later exposures to that environment elicit freezing, the characteristic rodent fear response: complete suppression of all movement except breathing and a stereotypic crouched posture. Time spent freezing indicates strength of memory for the context-shock association.

Liu et al. (2012) used a transgenic mouse line in which an excitatory opsin (ChR2) was expressed only during fear conditioning to the novel context, in the most active neurons in the dentate gyrus region of the hippocampus. Expression was limited by the tetracycline-controlled transcriptional activation system described above. ChR2 opens a direct channel for sodium (Na^+^) influx when photostimulated, to depolarize infected neurons. Hence only the specific infected dentate gyrus neurons most active during the context-shock pairing, which constitute the hypothesized cellular engram for the association, were reactivated by the photostimulation. Liu et al. (2012) ingenious experimental design was to reactivate the infected cells encoding the context-shock association to the first context while the animal was exploring a second and different novel context. No actual foot shock was administered in that second context. However, photostimulated experimental mice later demonstrated significant freezing to the second, non-shocked, context.

A follow-up study by Ramirez et al. (2013) in Tonegawa’s lab also expressed the opsin only in the most active dentate gyrus neurons, but this time during non-shocked exploration of the first novel context. They reactivated those specific neurons by photostimulation when the animals underwent context-fear (foot shock) conditioning in a second novel context. The experimental mice demonstrated the fear response when placed back in the first, neutral context, in which they never were shocked. These experimenters interpret their results straightforwardly: “we created a false memory in mice by optogenetically manipulating memory engram-bearing cells in the hippocampus” (Ramirez et al., 2013, p. 387).^11^

The Tonegawa lab’s second phase optogenetics hook experiments not only brought opogenetics to wider scientific notice, but also attracted huge public interest: ranging from *Smithsonian Magazine* to *The Guardian* to *Time Magazine* to neurogadgit.com, to name just a few popular sources that covered it. Google “false memory optogenetics” to get a quick feel for the number and range of popular media coverage. Its scientific revolutionary credentials were perhaps best expressed in these scientists’ own words. Noting that a case for the biological mechanism for a specific process involves three kinds of experimental evidence—“correlation” of the parallel occurrences of hypothesized mechanism and effect; “blockage” of the hypothesized mechanism, which demonstrates its necessity for the effect; and “mimicry” of the hypothesized mechanism, which demonstrates its sufficiency^12^—Ramirez et al. (2014, p. 3) note that “mimicry experiments for memory engram studies remained a considerable challenge”. Experimenters “widely recognized and agreed” that mimicry experiments were essential toward testing the cellular engram hypothesis. But “the lack of tools that could precisely label and control selected neurons involved in a particular memory posed formidable obstacles to carry out these experiments” (Ramirez et al., 2014, p. 3). Gene targeting techniques using microinjections of virus vectors for overexpressing genes like *CREB* and *α-CaMKII* in specific neurons prior to training had some limited successes (e.g., Josselyn et al., 2001, discussed above). Optogenetics, however, is the first tool which could reliably “selectively label and activate the memory engram-bearing cells to induce the predicted behavioral changes caused by learning,” and so reliably provide this final, essential kind of evidence for direct cell activity → cognitive function causal hypotheses. Experiments in the neurobiology of learning and memory could now reliably provide mimicry evidence. And they did, quickly. Two years after Liu et al.’s (2012) first results were published a review article appeared in *Trends in Neurosciences*, appropriately titled “The optogenetic revolution in memory research, ” which referenced 100 publications (Goshen, 2014).

## Concluding Remarks: Molecular Biology Vs. Physics as Paradigmatic Science

Real revolutions in contemporary cellular/molecular/behavioral neuroscience have little to do with the components and dynamics of Thomas Kuhn’s famous model, and mostly to do with novel tool development. The two concepts developed and illustrated here, motivating problem and hook experiments, are just first steps toward a metascience of experimental tool development, the key components of such scientific revolutions. They in no way exhaust all the interesting metascientific features of tool development in neurobiology, much less in science generally. Both components need additional support from other case studies. But those two alone show ways in which revolutions in neurobiology depart significantly from Kuhn’s model. It is also not clear how many other contemporary sciences share this revolutionary dynamic. But it is illuminating to note that cellular and molecular biology in general, and biochemistry, from which much of contemporary mainstream neuroscience stems, has now outdistanced physics as the most influential science of our time, in terms of research funding, number of publications, and number of practicing scientists. Obviously, numerous social forces drive a great deal of this current influence. But in light of it, one might legitimately wonder whether the still-lasting—and as I have argued here, mistaken—influence of Kuhn’s model of scientific revolution even on these fields is one lingering relic of mid-20th century philosophy of science, when physics was assumed to be the paradigmatic science, against which all others were compared and judged. Perhaps it is now time for cellular and molecular biology to assume that role? The recent growing influence of the “new mechanist” approach in philosophy of science, built most centrally on biology rather than physics, might be one important, albeit still implicit step in this direction (Machamer et al., 2000; Craver, 2007; Craver and Darden, 2014).

## Author Contributions

JB is the sole author of this manuscript.

## Conflict of Interest Statement

The author declares that the research was conducted in the absence of any commercial or financial relationships that could be construed as a potential conflict of interest.

## Footnotes

^1^These resources include: a set of textbooks that govern education into the discipline; a set of methods taught routinely to aspiring trainees, and researchers learning new techniques; a set of agreed-upon experimental results which inform new experiments (c.f. citation numbers for “classic” articles). What then explains the common impression, even among neuroscientists that no “governing paradigm” exists? Probably this attitude is due to the explicitly interdisciplinary scope of the field. Within given fields of neuroscience-certainly within cellular/molecular/behavioral neurobiology-many of Kuhn’s basic conditions on “paradigm” are evident; and I suspect this is true for other fields in the discipline with which I am less familiar. It is also worth noting in this content how vague Kuhn’s concept is Masterman’s (1970) well-known critical notice of Kuhn (1962) counted 21 distinct meanings in that work alone! I thank an anonymous reviewer for pushing me to clarify my quick dismissal pf neuroscience’s still being “pre-paradigmatic.”

^2^There is a genuine dispute about whether cognitive neuroscience armed with functional brain imaging methods is revolutionary. There is the well-known “computational” approach in cognitive (neuro-) science, and the many problems of consciousness. Perhaps the safest remark is that I am least confident that the argument to be developed in this article correctly applies to cognitive neuroscience. A serious investigating of this case is an important project for future metascientific research. I thank an anonymous reviewer for reminding me about these complexities.

^3^See Silva et al. (2014; chapter 3) for additional details of this case study and its aftermath, aimed at developing a metascience of Connection experiments.

^4^We will see this same feature of “motivating problem” illustrated in our second case study in the next section. However, it is an open and interesting metascientific question whether most tool development in neuroscience, revolutionary or otherwise, is guided by similarly structured motivating problems. Perhaps in some tool development histories all applications follow the tool’s invention. A broader metascientific study of tool development must address this potential alternate dynamic; suffice it to say that the two cases of revolutionary tool development discussed in this article answered to a recognized motivating problem. I thank an anonymous reviewer for mentioning this alternative account.

^5^An anonymous reviewer drew a potentially intriguing distinction between tool development, along the un-Kuhnian lines suggested here, and concept development in neurobiology, which might better match Kuhn’s account of paradigm shift and anomalies. I admit that my focus on the neurobiology of memory in these case studies might be occluding the discussion from conceptual revolutions, because plastic synapses have dominated theorizing and experimental research on memory and the brain for more than a century. This point is worth serious consideration, but would take this article beyond its intended scope of showing how central experimental tool development has been in neurobiological revolutions.

^6^Morris and Kennedy (1992) drew their title from this line of Alexander Pope’s early-18th century poem, “An Essay on Criticism.” The line of the couplet that precedes this quoted line, also quoted by Morris and Kennedy (1992) at the beginning of the review, is perhaps the most famous line from the poem: “A little learning is a dangerous thing”.

^7^We will also see this point illustrated in the second case study of neuroscience revolution in the next section.

^8^I will have more specific things to say in defense of optogenetics’ revolutionary nature after I explain the technique, canvass some results, and apply the notions of motivating problem and hook experiments to these details.

^9^See Silva et al. (2014; Chapter 1) for a broader perspective of how Tool Development experiments fit with other kinds of experiments in science. I thank an anonymous reviewer for reminding me about this concern, and for pointing me to the Otchy et al. (2015) article.

^10^Note that, intuitively, the motivating problems for each of the other two instances of revolutionary tool development in contemporary neuroscience I mentioned above-high-output electrodes for single-cell recording *in vivo* and functional brain imaging -also share this feature. One might be starting to sense a pattern …

^11^Philosopher Robins (forthcoming) makes interesting use of these and other results from the Tonegawa lab to challenge “constructionist” views of memory, now prevalent in psychology and cognitive neuroscience.

^12^These labels are from neurobiologist Sweatt (2009). The attendant concepts are similar to Silva et al. (2014) notions of “non-intervention,” “negative manipulation,” and “positive manipulation” experiments, which combine to make up “Convergent Three integration,” although there are subtle differences, especially concerning “mimicry” and “positive manipulation.” That discussion is beyond the scope of this essay.
